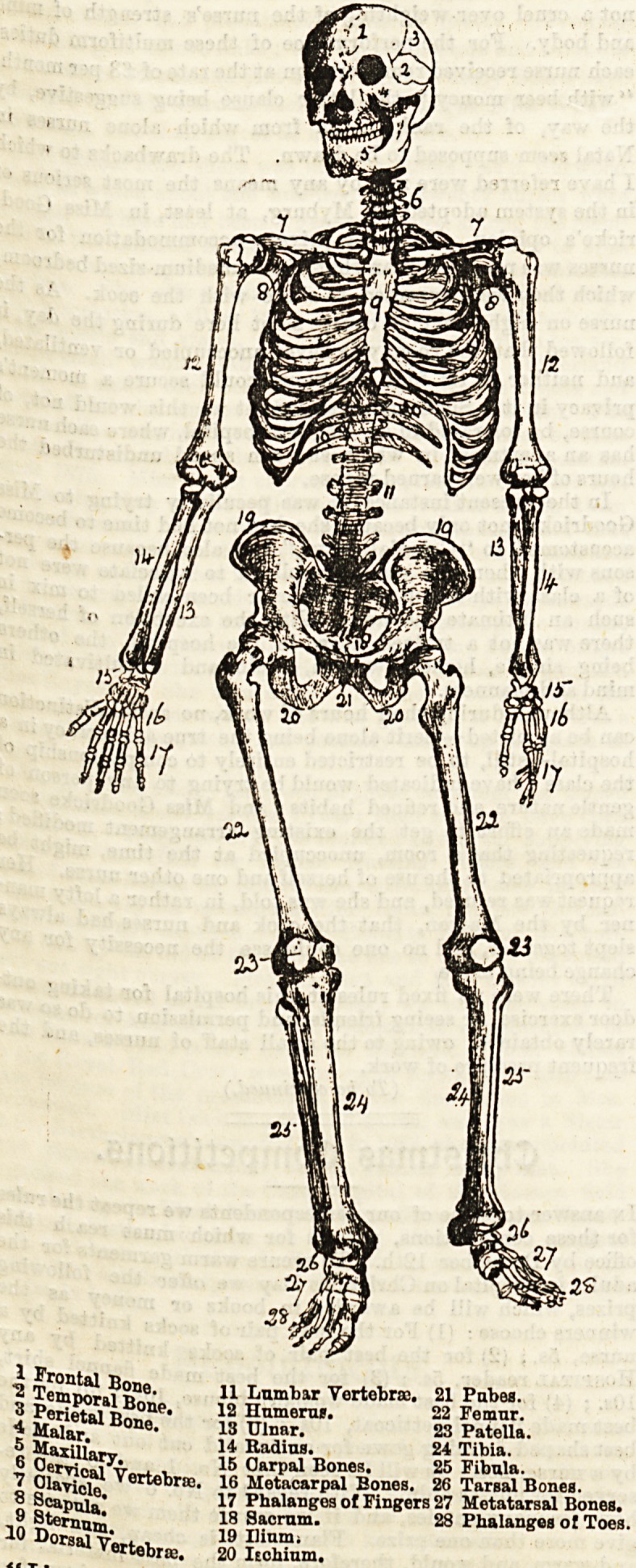# The Hospital Nursing Supplement

**Published:** 1891-11-07

**Authors:** 


"The Hospital" Nursing Mirror.

Being the Extra Nursing Supplement of "The Hospital" Newspaper.

Contributions for this Supplement should be addressed to the Editor, The Hospital, 140, Strand, London, W.C., and should have the word "Nursing" plainly written in left-hand top corner of the envelope.

### En Passant.

SHORT ITEMS.—Miss Reid, Queen's nurse at Rugby, attended 143 cases last year; the Committee report their appreciation of her work.—Dundee now boasts six district nurses who, in twelve months, have attended 602 patients. The home needs enlarging and further subscriptions are needed as the work increases so rapidly.—The St. John's Community is no longer responsible for the nursing of the Metropolitan Hospital.—The patients in Liskeard Infirmary are now well cared for by Nurse Dromgoole, and old stories are to be buried.—Nurse Rosa Francis has been committed for trial on the charge of having stolen jewellery from the late Mr. Willard, while engaged in nursing him on his death-bed.—The prettiest Christmas picture this year is that given away with "Yule Tide;" it is a copy of Henrietta Rae's "Lady with the Lamp," otherwise Miss Nightingale at Scutari.—Mr. Mackeson is the student who is so kindly arranging entertainments for the nurses at Guy's.

TOMBSTONES.—An Irish lady writes to one of the papers a laudatory notice of the Brassey Holiday Home, where she describes herself as dawdling away her holidays with the greatest happiness. She concludes: "Yesterday we went on a picnic to Bexhill, and enjoyed ourselves thoroughly. We visited the old church, and spent a long time amongst the tombs. I enjoy reading inscriptions, they show such variety of mind and temperament, and one can almost tell the style of inscription to be met with from the look of the headstone. By-the-bye, my wanderings among the tombs led me to a very sad conclusion. I suppose you will say that is only what you expected, but I allude to the Pension Fund. It has made no arrangements for burying us or providing us with stones; but I suppose it thinks—and possibly it is right—that a nurse should be content with a nice green mound, Nature's true covering." Certainly we think a nurse might be better employed than in saving up for, and thinking about, her tombstone.

AMERICAN GOSSIP.—"So you are going to have a bed for a sick nurse? You will find it very useful; we have one in the New Polyclinic Hospital, Philadelphia, endowed by Mrs. Ingersoll, and known as the Ingersoll Bed. The nurses of the Beneficial Association furnished the room in which the bed is. I am now in Atlantic City with a patient; it is a wonderful wooden city on the sea, with a broad walk five miles long all along the coast. We have had a hot dry summer (the opposite to yours) the thermometer at 90° at midday, and never below 70° at night. I am glad to hear of the success of the Co-operation, and hope you will see that the rules are strictly adhered to; having worked so long under a directory I know that success and strictness go together. Miss Sarah Sheldon, head of the Buffalo Directory for Trained Nurses, has been taking a long holiday, one of her sisters doing her work in the meanwhile. A native of Finland has lately started some classes of Swedish Gymnastics at Buffalo. A very remarkable case, exciting much attention, is that of Miss May White, of Meadsville, who has slept almost continuously since June 21st, when she had a fall and injured her spine. At first it took five hours to rouse Miss White, so she was only roused once a day; now the doctor can rouse her regularly three times a day for food, and he hopes to secure her recovery in time. He ascribes her malady to uræmic absorption. Dr. Weir Mitchell (a true admirer of trained nurses) was President of the Congress at Washington, which Prof. Gairdner, Doctors Ord and Batty Tuke, and Sir William MacCormac attended."

TAUNTON JUBILEE INSTITUTE.—The report of the Somerset Hospital and Jubilee Institute just issued tells of a staff of twenty nurses and probationers doing good work. The classes for nurses have been continued by the House Surgeon and Lady Superintendent. The Committee have been able, during the year, to meet eighty-nine applications for nurses in private families, and have supplied eight extra nurses to the hospital. The Committee being strongly of opinion that it was most desirable to find some sort of recreation for the nurses off duty, a piano has been provided for the nurses' sitting-room, and is thoroughly appreciated. The need for a quarantine ward, to which reference was made in last year's report, is still urgent. The Committee wish to express their appreciation of the conscientious efforts of Miss Macdonald whilst Lady Superintendent, and they have every confidence in the appointment of Miss Wilson in her place, she having already shown great organising powers.

GLASGOW AGAIN.—In a signed letter just received, a correspondent writes us *re* the Glasgow row: "Go where you will a nurse is not treated as she deserves to be. Her lot is nothing but one continuous round of sicking (?) contention. She is often treated like the chimney sweeper, &c." We really cannot quote more of this untruthful rubbish, but what a shocking and disgraceful thing that women should permit themselves to write and believe such statements! We can only say that we wish such women would take their cold dinners so seriously as to immediately and for ever leave the infirmary, where their presence in the uniform of a servant of the sick is a horrible anomaly. The good nurses do not want selfish grumblers in their ranks; go and get hot dinners elsewhere you grumblers and leave the "continuous round of sicking contention," to those who find it the noblest and best existence on earth; the one profession which calls forth all the good qualities of a good woman, and makes her completely happy because it grants her the privilege of labouring "as Jesus did, for men."

THE EASTERN HOSPITAL.—Dr. Collie has at last sent in his resignation, and the Matron has sent in hers at the same time. Let us hope the Board will now put the Matron in complete control over the nurses, and see that the Medical Superintendent is not allowed to contradict the Matron, when, for instance, she objects to nurses dancing in a ward. Of Dr. Collie, as a medical man, many speak well, but as a Superintendent he was certainly a failure, as the whole character and tone of the nurses brought forward as witnesses in his favour show. There ought to be a clean sweep at the Eastern Hospital and a fresh start. The attempt of the lay press to whitewash Dr. Collie and make the Board retain him in his position is extraordinary, especially after Dr. Collie's undignified delay in sending in his resignation and his present plea to the Board for a pension. The whole story is one more proof that it does not do to put a doctor directly over nurses, and that the Matron ought always to have supreme control. In the first case the doctor is always liked by some, and equally disliked by others of the nursing staff; presumably he is apt to indulge in favouritism. The fate of Dr. Collie brings back the thought of Dr. Conry and the Hope Hospital scandals; some of our readers may be interested to hear that Dr. Conry holds a lucrative post in Africa, and is there enjoying an adventurous and interesting existence, equally free from guardians and nurses.

### Lectures on Surgical Ward Work and Nursing.

By Alexander Miles, M.D. (Edin.), F.R.C.S.E.

Lecture XXXVIII.—(*continued*).

*Gouges* are used to clip away pieces of bone, diseased or otherwise, or in opening the skull in some cases. They are simply chisels with grooved blades, and are of various sizes.

*Trelat's gouge* is straight, with a long metal handle; while others have ebony handles.

*Gimlets* are used in surgery to pierce bones preparatory to wiring fragments together, for example, after fractures, and they should have an eye near the point, so that they may carry the wire through.

*Mallets* are used along with the gouges or osteotomes. These may be of steel or of wood.

Sharp Spoons or Scoops.—These are often found useful in dealing with sinuses associated with diseased bone, and even in scraping the bone itself. They were originally used by Volkmann, and often called by his name. In some the spoon is round, in others oval, and the edges are just sufficiently sharp to remove diseased tissue, sparing that which is healthy. Preferably, the whole instrument is made from one piece of metal, so that it may be sterilised by heat without being damaged. This is the more necessary, as the sinuses, &c., in which it is employed, so often swarm with septic and other organisms that there is danger unless great precautions are taken. *Lister' spoon* is a much shorter and stouter instrument, but otherwise resembles that of Volkmann. Other sharp spoons, much smaller than the above, are used in the treatment of lupus, where it is necessary to scoop out the contents of each tubercular nodule. These are sometimes called *lupus curettes*.

*Directors* are narrow, blunt pointed instruments about six inches long, furnished with a deep groove down the centre, along which a bistoury may be passed, and the extent and direction of an incision thus be accurately determined. The above represents the *ordinary director* used in opening up a sinus, or in opening an abscess by Hilton's method. At one end it is hollowed out so as to form a blunt spoon or scoop. *Spence's Hernia director*, used to guide the knife to the constricting band in the operation for strangulated hernia, is about three times as broad as the ordinary director, and the groove is not so deep. *Key's Hernia director* is also broad, with a shallow groove, and in addition has a distinct curve on the blade. *A German silver director*, probe-pointed, is sometimes used to guide the knife in slitting up a fistula-in-ano.

*Retractors* are used to separate the lips of a wound during an operation; to hold aside, and so remove from danger, important structures, such as large vessels or nerves, or to steady a tumour while being dissected out. The simplest form of retractor is a short, broad sheet of copper, which may be bent so as to form a hook of any size. Another is made of wire which will also bend to any desired angle. Various forms of steel hooks are also used, the best of which perhaps go by the name of Ollier. They are made in sets of different sizes, and have several blunt teeth.

*Dressing Forceps* are of various patterns, *e.g.*, which resemble artery forceps, but want the catch on the handles.

Others resemble polypus forceps, but are serrated only half-way up the blades.

*Sinus Forceps* have long, narrow, tapering blades, serrated at the point for a very short distance. They are used to pick out small substances, such as fragments of dead bone from a narrow, deep sinus, or for introducing drainage tubes into a deep wound, care, however, being necessary lest the sharp points damage important structures. They also serve to introduce small fragments of silver nitrate into the bottom of long sinuses which are obstinate in healing.

The illustrations are used by kind permission of Messrs. Maw, Son, and Thompson, and Messrs. J. Weiss and Sons.

### Notes and Queries.

To Correspondents.—1. Questions or answers may be written on post-cards. 2. Advertisements in disguise are inadmissible. 3. In answering a query please quote the number. 4. A private answer can only be sent in urgent cases, and then a stamped addressed envelope must be enclosed. 5. Every communication must be accompanied by the writer's full name and address, not necessarily for publication. 6. Correspondents are requested to help their fellow nurses by answering such queries as they can.

### Query.

(12) Is there any home where a poor incurable woman could be received free of charge, and where votes are not necessary?

### Answers.

*Christmas Competitions.*—Parcel received from Mrs. Fleming.

(6) Salts of lemon will remove iron-mould stains from linen. Solution of chloride of lime will remove tea and coffee stains, and perhaps iron-mould stains as well.

(9) *Chilblains.*—Paint your chilblains with white iodine; take as much exercise as possible, and do not sit over the fire.

(9) *Chilblains.*—Keep your feet from the fire as a preventive. As a cure paint with iodine. There is colourless iodine for the hands.

(10) Apply to Miss Lobb, Children's Home, Lambourne, Essex.

*Nurse E.*—See answer to (9).

*Nurse G. P.*—See our advertisements for a list of books on nursing; Domville's "Manual," Lewis's "Textbook," and Lücke's "Lectures" are all excellent.

*Another Old Nurse.*—You do not send name and address, and we never pay attention to anonymous contributions.

*N. A.*—Messrs. Southall, of Birmingham, will supply you with a wicker basket, fitted for district work, for 30s., or with a morocco bag, fitted, for two and a-half guineas.

*J. G.*—You persistently write on both sides of the paper, that is why your queries, &c., are "snubbed," as you term it.

*Ignoramus.*—Certificates need not be printed on parchment, but it is better to do so, as it is more durable than paper or card.

### Examination Questions.

TWENTY-EIGHT drawings of skeletons were received in answer to last month's question, and the prize was awarded to Miss Annie Mills of Walsall, for the drawing reproduced. Most of the drawings were beautifully done, and the following deserve honourable mention: Nurse Ellinor Smith, "Nurse Lee," Nurse Mumford, Nurse Doughty, Nurse Brittan,

| | | |
|---|---|---|
| 1 Frontal Bone. | 11 Lumbar Vertebræ. | 21 Pubes. |
| 2 Temporal Bone. | 12 Humerus. | 22 Femur. |
| 3 Perietal Bone. | 13 Ulnar. | 23 Patella. |
| 4 Malar. | 14 Radius. | 24 Tibia. |
| 5 Maxillary. | 15 Carpal Bones. | 25 Fibula. |
| 6 Cervical Vertebræ. | 16 Metacarpal Bones. | 26 Tarsal Bones. |
| 7 Clavicle. | 17 Phalanges of Fingers | 27 Metatarsal Bones. |
| 8 Scapula. | 18 Sacrum. | 28 Phalanges of Toes. |
| 9 Sternum. | 19 Ilium. | |
| 10 Dorsal Vertebræ. | 20 Ischium. | |

"Liverpool," and Nurse Headford. The last had drawn the skeleton in profile with extended arm, and in common with other competitors, had thus exceeded the three inches breadth. The names of all competitors have been entered for the prize for industry; will those nurses who have adopted nom-de-plumes please retain them for the six months? We now offer an illustrated book on Obstetric Nursing for the best notes of an interesting medical or surgical case neatly chronicled in "The Nurse's Case-book." The actual leaves of the case-book must be sent, for we will, if possible, reproduce the prize record. The two chief points noted in giving the prize will be that the case contain some typical or interesting feature, and the neatness and conciseness of the notes. Answers must reach THE HOSPITAL, 140, Strand, London, not later than December 1st.

### For Reading to the Sick.

### SELF CONTROL.

IT is easy to give way to natural desires and fancy we cannot do anything that is troublesome or against the grain, and then in time we lose the power of saying "No!" either to ourselves or other people, and we become as reeds shaken by the wind.

Not that there is any merit in opposition for the sake of opposing; on the contrary, a pliable temper is a very great blessing; but we should try and hit that happy medium of being neither too stern nor too complaisant, which is really self control. Those who have exercised it more or less all their lives, find the value of it when they are lying on a sick bed.

To be able to bear agony with patience, to suppress our groans and sobs for the sake of others, to bear the numberless trials of illness with submission and even cheerfulness, are the graces of those who have fought with themselves and conquered. They have crushed their bad tempers, repressed their burning desire to make everyone bend to their will, they no longer struggle against moral restraints, and now reap the benefit. They do not ask why they are tied down to the couch of pain, while others walk about whole; it is enough that God has thought fit to afflict them, and they murmur, "Thy will be done."

But how are we to gain this self control? you say. By comparing our lives to that of the Man of sorrows, and seeing how near we resemble Him. "Learn of Me," He says, "for I am meek and lowly of heart, and ye shall find rest unto your souls."

"Christ pleased not Himself," says St. Paul. Shall we then expect to have nothing but smooth things for our portion? If He bore hunger and thirst and pain, the contempt and scoffs of His enemies, what are we that we should expect to be free from trouble and suffering? These very sufferings will draw us to Himself, and with the utmost love He will not only teach us to bear them, but our wills being under control He will bear them for us. His gracious promises fall on our hearts like the shadow of a great rock in a weary land. He beseeches us, "Come unto Me all ye that labour and are heavy laden, and I will give you rest." Thus shall we possess our souls in patience, because His strength is sufficient for us. We will go on then humbly in the footsteps of Christ, and the road which seemed steep and thorny will become smooth. Our exertions will strengthen our will; we shall climb higher and higher till we leave earthly cares and troubles far behind us, and bask in the light of the land which now often seems very far off. For it is not license which brings happiness in either this world or the next, but the keeping under our bodies, and bringing every thought into subjection to Christ.

---

A FRENCH doctor recommends vaccination with steel pens. Presumably he does so on the ground of economy, as owing to their cheapness a fresh pen can be used for each operation, and danger of infection from the lance is thereby avoided.

### A Nurse in Natal.

A very few days experience of her new surroundings sufficed to prove to Miss Goodricke the great dissimilarity between an English and colonial hospital.

She found that not only were the discipline and management very lax, but that the ordinary rules of an English hospital were either unknown or disregarded. A short description of the routine observed in this establishment may not be uninteresting to English readers. A Matron and a dispenser presided over the internal economy of the hospital, and under their control was a nursing staff consisting of two day nurses, and *one* night nurse only. There was a white female cook, and a small number of Kafir and coolie men attached to the institution. There were no probationers, and no wardmaids as in an English hospital. The Matron had been married in early life to Admiral Fraser, but having been soon left a widow, with very small means, she had obtained and filled for many years this post in the Myburg hospital. Though a kind and good-natured person, and well-fitted to shine in social circles, this lady had had no previous knowledge or experience of hospitals and nursing. Whatever natural gifts of management or housekeeping she might have, it could not therefore be expected that she should possess that thorough acquaintance with the work and training of hospital life which, in England, would be considered absolutely indispensable to her position. As there were no wardmaids attached to this institution, the rougher kinds of work usually devolving upon them, was here performed by the coolie and Kafir servants, the latter scrubbing the floors, &c., and the former attending to those private details of the sick ward, which, in England, it is the nurses' special duty to superintend. The two day nurses, whose hours were from six a.m., to nine, and sometimes ten p.m., were expected, in addition to the usual work in the wards, to fold the clothes from the wash, to see them mangled by the coolies, and to put them away in their respective places. When it is considered what a large amount of linen of various kinds must be in use in even a small hospital, it will be readily conceived that this must have been no slight addition to their nursing work.

While on day duty, they were desired to fill alternately the posts of "House Nurse" and "Kitchen Nurse"; it being the duty of the latter to prepare the breakfast for each patient in the hospital, and send it in by a coolie. Dinner, though cooked by the cook, was carved and distributed by the nurse; and the evening meal was again prepared by her in like manner with the breakfast—the cook being only expected to boil the water with which each patient's tea or cocoa was made. The whole of this kitchen work was performed in one detached room, about fifty yards from the main building, to which there was no covered way, so that the nurse was exposed to the weather at all times. Any one at all acquainted with the routine of English hospitals, will at once recognise the difference between the work expected of the nurse in this colonial institution, and that considered consistent with her duties and character in England. And though Miss Goodricke had the good sense to feel that she must not expect the same conveniences and comforts, or the perfect organisation to be met with in the large and well-endowed hospitals of this country, she could not but think that there was here great room for improvement. In a hospital containing at times as many as forty patients, with a staff of only two day nurses, could it be expected that each or either of these could give to her patients that unremitting attention so absolutely necessary while her time and thoughts were divided by such different duties in a distant part of the hospital? Such an arrangement was certain to produce either a lessening of interest in her nursing work, or else an irritation and worry of mind prejudicial both to her patient and herself. If, however, the duties of the day nurse were many and varied, her task was still more onerous and trying when it became her turn to go on night duty, for she was then the only one on whom depended the care of every patient in the hospital; and even in the case of a delirious fever patient, the only assistance she could command was that of a Kafir or coolie who slept upon the premises. Let those who know what it is to watch through the long hours of night beside one sick bed, decide if this were not a cruel over-weighting of the nurse's strength of mind and body. For the performance of these multiform duties, each nurse received remuneration at the rate of £3 per month, "with beer money," the latter clause being suggestive, by the way, of the rank in life from which alone nurses in Natal seem supposed to be drawn. The drawbacks to which I have referred were not by any means the most serious of in the system adopted at Myburg, at least, in Miss Goodricke's opinion. No other private accommodation for the nurses was provided than that of one medium-sized bedroom, which they were expected to share with the cook. As the nurse on night duty of course slept here during the day, it followed that the room was never unoccupied or ventilated, and neither of its four occupants could secure a moment's privacy in it. Such an arrangement as this would not, of course, be tolerated in an English hospital, where each nurse has an apartment in which she can spend undisturbed the hours of her well-earned repose.

In the present instance it was peculiarly trying to Miss Goodricke, not only because she had not had time to become accustomed to "Colonial ways," but also because the persons with whom she was now obliged to associate were not of a class with which she had ever been called to mix in such an intimate manner. With the exception of herself, there was not a trained nurse in the hospital, the others being simple, homely women, plain and uncultivated in mind and manners.

Although during their hours of work, no social distinction can be admitted—merit alone being the true aristocracy in a hospital—still, to be restricted entirely to companionship of the class I have indicated would be trying to any person of gentle nature and refined habits; and Miss Goodricke soon made an effort to get the existing arrangement modified; requesting that a room, unoccupied at the time, might be appropriated to the use of herself and one other nurse. Her request was refused, and she was told, in rather a lofty manner by the Matron, that the cook and nurses had always slept together, and no one could see the necessity for any change being made.

There were no fixed rules at this hospital for taking outdoor exercise, or seeing friends, and permission to do so was rarely obtained, owing to the small staff of nurses, and the frequent pressure of work.

(*To be continued.*)

### Christmas Competitions.

In answer to some of our correspondents we repeat the rules for these competitions, parcels for which must reach this office by December 12th. To secure warm garments for the adults in hospital on Christmas Day we offer the following prizes, which will be awarded in books or money as the winners choose: (1) For the best pair of socks knitted by a nurse, 5s.; (2) for the best pair of socks knitted by any Hospital reader, 5s.; (3) for the best made flannel shirt, 10s.; (4) for the best made woman's blouse, 10s.; (5) for the best made flannel petticoat, 10s.; (6) for the best made and best shaped dressing gown for an invalid cut out and made by a nurse, 20s. It will be seen that No. 1 and 6 are reserved for nurses only. With regard to No. 6 we specially hope for many entries, and if we secure them we propose to give more than one prize. Flannelette is cheap, and light, and warm, and would, therefore, form the best material for the dressing gown. In judging, four marks are given for workmanship, four for shape, and two for general appearance; therefore, it is not wise to spend time on elaborate trimmings. Long seams may be done by machine. Parcels of clothing for distribution, but not for competition, will be gladly received and acknowledged in these pages.

### Presentations.

Miss Dunstan, the Matron of the Royal Albert Hospital, Devonport, who has resigned after five years' service, was on her departure from that institution last week presented with a pair of very handsome ivory-backed brushes and comb by some members of the nursing staff. She also received some gifts as tokens of esteem from several friends.—Miss Wilkinson, on her departure from Derby was presented by the Sisters, nurses, and probationers with a pretty travelling clock and gold sleeve links; and by the porters and servants with a silver teapot and jam spoon. The Weekly Board have recorded in their minutes their appreciation of Miss Wilkinson's work and their regret at her departure.

On October 30th there was an "At Home" at 3, Burwood Place, at which about one hundred and twenty nurses were present. There had been a general desire among the members of the London Association of Nurses to convey to Miss Walker some expression of their thanks for her valuable services. The gift consisted of a silver-mounted oak tray, with inscription, a silver tea-pot, cream jug, and sugar basin, brass kettle and stand, a white and gold porcelain tea service, a small walnut table, and a larger walnut Sutherland table. A small book contained the names of the donors and included all the nurses belonging to the Association. The presentation was made on behalf of her fellow nurses by Miss S. A. Payne, who has been connected with the Association since 1875. Miss Payne expressed the pleasure they all felt in knowing they would still have Miss Walker among them for the future. Miss Walker in returning thanks said that she was very much touched by their expressions of kindness and good-will, and felt that the success of her work had depended on the hearty co-operation and the spirit of loyalty towards each other, which she had always observed among the nurses. She had also to acknowledge the support and help of others who had acted with her, particularly the Misses Briggs and Miss Rayner. She also asked that they would extend the same treatment (for which she was grateful to Miss Firth's niece, Miss Sprigg, who has recently taken up her place as Superintendent.

We gave the names last week of those on whom the Queen has conferred the Royal Red Cross; their services have been as follows:—Lady Roberts has founded a fund for supplying homes in the Hills for Indian Nursing Sisters, and has opened such homes at Murra and elsewhere; also in 1889 there was a cry for more nurses for India, but the Government would not hearken. Lady Roberts took up the subject, and by the aid of the fund she has so cleverly organised sent out eight nurses. Mrs. Damant and Mrs. Cawley cared for the wounded under fire in India; we told the story in our issue for August 8th. Mrs. Damant is a connection of Miss Wilson's and is now living in England. Their claims to the Royal Red Cross were brought forward at this late date because of the presentation of the decoration to Mrs. Grimwood. Miss Loch is a trained nurse, and was a Sister at St. Bartholomew's hospital till, in 1888, she was appointed first Sister-Superintendent on the Indian Army List. She organised the work of the Case hospital of the Hazara field force during the Black Mountain Expedition. Sister Welchman and Sister Lickfold are two of Miss Loch's original staff, and have worked in India for three years. It will be noted that all six recipients of the Cross have received it for service in India.

### Appointments.

Birkenhead Fever Hospital.—Nurse Yeomans has been appointed to the not too enviable post of Matron at the above Hospital. She trained at Park Hill and Grafton Street, Liverpool, Fever Hospitals, and holds excellent testimonials; we are sure she will do good work at Birkenhead, and we give her hearty sympathy and wish her success.

Derby Infirmary.—Miss Jessie F. Parsons has been appointed assistant Matron of this Infirmary. She trained at the Wolverhampton Hospital, and has been working at the Bolton District Nurses' Home. She holds the L.O.S. diploma and excellent testimonials.

Correction.—Miss Rimington has been appointed Sister, not Matron, at Ingham.

### Everybody's Opinion.

*[Correspondence on all subjects is invited, but we cannot in any way be responsible for the opinions expressed by our correspondents. No communications can be entertained if the name and address of the correspondent is not given, or unless one side of the paper only be written on.]*

### MONTHLY NURSES.

"Another Monthly Nurse" writes: I am very sorry to read in the letters of "S." and "A Monthly Nurse" that they got so little teaching and practice for their training fee, and consider that when a proper amount of knowledge cannot be imparted during a residence of eight weeks, the pupil should be allowed to remain longer in the hospital without paying an extra fee. Surely there has been a change for the worse in these respects since I trained in London early in 1882. I am now writing with my little note-book for that year open before me. I was comfortably lodged and properly fed, but found the work very hard. In all, twelve cases passed through my hands, viz., I was present and assisted at six confinements, four of which cases, with their infants, I nursed for a fortnight, two at a time. Next came three patients with their infants, who for some reason had been transferred from their own nurses after being confined over a week. These remained with me nearly a week longer, and after this my remaining time was occupied for twelve hours out of each twenty-four—sometimes by day, sometimes by night—in relieving nurses who were in charge of serious and anxious cases. On one point, and one only, I left my hospital dissatisfied. I had expected a certain amount of practical teaching as regards the actual delivery of the woman. This was kept exclusively for the pupil midwives. I could not afford a further fee of 26 guineas, so supplemented my nursing course by some private lessons taken under an experienced midwife, and for these I have, in several emergencies, had good reason to be thankful. I should like to mention that I believe much of my success is attributable to the fact of my having, prior to becoming a monthly nurse, earned my living for thirteen years, first as under and then for nine years as head nurse in the nursery. I lived in good families, took charge of my babies day and night, prepared everything for each new arrival, and on several occasions assisted the monthly nurse. I do not think I could possibly have had a better preparation than this. A lady's ways, and the management of an infant according to a lady's ideas were quite familiar to me. The need of some such knowledge is glaringly apparent in some nurses of the present day, and is one cause of their failure. There is certainly room for improvement in this matter, and it appears to me that what is really needed is—First of all, stricter inquiry as to the actual suitability of the candidate; secondly, longer and fuller training for the same fees, viz., instead of ten guineas for eight weeks, or fifteen guineas for twelve weeks, say ten guineas for twelve weeks. This, with extras for lectures, washing, and breakages, is quite expensive enough. Lastly, a proper examination of the finished pupil by the medical staff before granting the certificate. Surely this might and ought to be done. We should then be protected against the inefficient monthly nurse, who, on finding herself a failure in her own line, tries to make up for her losses by poaching on the preserves of our medical and surgical sisters.

### MALE NURSES.

"A Witness" writes: It is scandalous how male nurses are treated at the Hamilton Association. While many nurses have had to remain at home for want of cases several weeks at a time, the management have kept on advertising for more nurses, not that they wanted them, but that they might raise a few pounds in entrance money, all nurses now accepted having to pay twenty shillings entrance fee, which goes towards keeping up the place. Besides, fresh nurses help the funds in another way; they are paid two shillings per day, work or play, for the first six weeks, but care is taken to keep them in employment, which pays the Association from four to five shillings per day, but since the place was first started there has been very little real concern shown for the nurses; self has been present in nearly all the alterations that have taken place, which have been numerous and mostly one-sided. There is always more concern about the employer's five shillings engagement fee than there is about the nurse's rate of wages, for whether a nurse is paid one or four guineas, the engagement fee is five shillings in either case, so that get the case, which means five shillings, and the wages are arranged afterwards, not often in accordance with the experience of the nurse, but what the employer likes to offer. More could be said, especially about the absurd secrecy that is carried on, for strangers know more about the place than the nurses do; but, in conclusion, let me mention that many of the fresh hands, with many years' nursing experience, have to undergo the two shillings per day system already referred to. As they are fully qualified, why not let them commence as such, for it is absurd to make them undergo probation.

[The Secretary of the Hamilton Association informs us that he is not now advertising for any more nurses, and no extra nurses have ever been taken on unless it was thought employment could be provided for them. It has always been the practice to send the older nurses to the most important cases. We are afraid "A Witness" is one of the grumblers.]

### Wants and Workers.

*The District Nurse*, 82, Victoria Street, Ashton-under-Lyne, returns her sincere thanks for old linen and stockings from Mrs. W., of Brixton.

*The Lady-Superintendent, West Norfolk Hospital*, King's Lynn, thanks "C. S." for bed-jackets, and "Emma" for scrap-book and cards. Ward table-cloths and Christmas gifts for the patients would be very welcome.

*Sister Garrard* will be grateful if anyone will send any old perambulator for the use of the babies at the Children's Home, Lambourne, Essex.

### Bruce.

He was a gentleman by birth; you could not mistake that. All his points were perfect, and so was his temper.

Any dog is good company—always supposing he is thoroughbred; you can not well train a cur, it is almost impossible; there is always the chance of some mean trait sneaking out unexpectedly—but the best companions, caninely speaking, are St. Bernards, Newfoundlands, and mastiffs. Bruce was a mastiff; I owned him from his birth, and I knew his pedigree thoroughly. Such a big-headed, awkward-legged, gambolling pup as he was.

Bruce was like a baby to Mary and me. We had no children, but while we had Bruce, we never seemed to miss them; we taught him endless tricks, and made a companion of him in every way. We were as poor as a couple of church-mice when we married—country doctors are usually—but we were happy. I got a village practice in a thinly populated district, where we started merrily enough on our life-journey. But the place was a doleful one, and as the years went by, its dreariness began to tell on Mary; she grew peaky and nervous, and would start, in affright, at her own shadow, as it were. If it had not been for Bruce's protection, I do not believe she would have been able to cross the moorland alone. A Londoner by birth, she never took honestly to the life of the country, and of course she had to be a good deal alone; my widely-spread—in a sense—practice filled up most of the hours of the day.

"Never go out without Bruce, my dear," I enjoined, and she promised, so my mind was at ease about her, for I had carefully trained the dog to be able to "go for" a man scientifically, in this way. I constructed a dummy, and by patient teaching had instructed Bruce how to set upon a foe; he being good tempered and obedient, I soon made him comprehend that so long as the dummy never moved he was only to watch it; but let it attempt to move, as I took care to make it do occasionally, by means of long ropes attached to it, then Bruce was trained to pin it down gently by the throat *only*. If the dog were rough or tore at any other part of the body save the throat, I whipped him severely.

"Its horrible, simply horrible to teach him that work;" said Mary shudderingly, "Supposing that dummy were real —a living man!"

"Exactly! Suppose it were, and suppose that in my absence miles away, the living dummy arrived, and came down upon you for your purse or your watch, you'd look rather foolish, ma'am!"

"Oh!" but still Mary looked incredulous.

It was late October. The winter glooms was creeping upon us; each evening was treading more swiftly on the heels of the afternoon. Jogging home from a monotonous round, I pulled up my coat-collar as I met the chilly wind crossing the moorland, and devoutly hoped that Mary was safe at home in her warm, little drawing-room, for it had been an eerie afternoon, and she was atmospherical.

As I left the moor, something blue under a pine-tree, on its outskirts, attracted my eye. I should not have to pass the object, but in a curious, inexplicable way, I was drawn as if by invisible cords to turn my horse s head towards it. I have, rightly or wrongly—let thers decide—made it a rule to give myself up to that sort of magnetism which is to us so mysterious in its compelling power. The few occasions in my life on which I have resisted its influence have been disastrous for me.

As I drew up close to the belt of pines, rather than a wood, and saw that the something blue was the fur-lined cloak Mary had told me, that morning, she intended bringing out of her wardrobe, in obedience to the chilly hints October was throwing out of coming winter. I then knew why I had been thus led. With a beating heart I sprang from the trap, and clearing the bushes between us was soon bending over my wife's figure. Crouched on the ground close up against the tree, cowered Mary, with closed eyes. She had not fainted, I saw at a glance, but she was certainly helpless from fright, and could neither speak nor move. Bewildered, I looked round for a cause, and then, for the first time, saw, at the distance of a few feet, a spectacle that will never fade from my memory.

Stretched out upon the dry carpet of pine needles lay the figure of a man, with face upturned to the dark canopy of the tree-tops. He was motionless as death; and well he might be, for, by his throat, Bruce had pinned him to the earth.

But—but what was *that?*

With eyes dilated in horror, I gazed upon a thin, red stream creeping slowly, slowly nearer over the crisp, dry needles, staining them as it meandered along.

*(To be continued.)*

### A Bed for a Sick Nurse.

Our scheme has received great help this week from Nurse Elms, who has collected for it the handsome sum of £7 12s. as follows:—Mrs. Henry E. Foster, 3 guineas; Mr. C. Bailey, Miss Bailey, Mr. Lillitoe, and Miss Blanche Welb, 10s. each; Mr. H. Welb and Mr. H. B. Welb, one guinea each; Nurse Clarke, 1s.; C. Gregory, 1s.: and Nurse Elms, 5s. We also have received from A. E. S. and A. G., 2s.; L. J., 1s.; One of the Second Thousand, Dundee, 3s.; A. E. C., 5s.; Nurse Mary, 2s.; Nurse Gerrard, 2s. 6d.; One of the First Thousand (Uppingham), 5s.; making a total of £24 18s. and leaving six guinea subscriptions still needed. Are no hospital committees going to follow the example of the Macclesfield Infirmary Committee and send us a guinea?

### Amusements and Relaxation.

### SPECIAL NOTICE TO CORRESPONDENTS.

**Fourth Quarterly Word Competition commenced October 3rd, ends December 26th, 1891.**

Competitors can enter for *all* quarterly competitions, but no competitor can take more than *one first prize* or *two prizes* of any kind during the year.

Proper names, abbreviations, foreign words, words of less than four letters, and repetitions are barred; plurals, and past and present participles of verbs, are allowed. Nuttall's *Standard* dictionary only to be used.

The word for dissection for this, the SIXTH week of the quarter, being

"GUY FAWKES."

| Names. | Oct. 29th. | Totals. | Names. | Oct. 29th. | Totals. 128 |
|---|---|---|---|---|---|
| Lightowlers | 89 | 201 | Jenny Wren | 77 | 203 |
| Bonne | 86 | 223 | Darlington | 77 | — |
| Morico | 98 | 239 | Nurse G. P. | — | 123 |
| Robes | 51 | 143 | Hetty | 65 | 174 |
| Dulcamara | 94 | 211 | Janet | 71 | 145 |
| Psyche | — | — | Jackanapes | 69 | 99 |
| Agamemnon | 92 | 226 | Ex Nurse | 48 | |
| Nurse J. S. | 83 | 203 | | | |

### Notice to Correspondents.

All letters referring to this page which do not arrive at 140, Strand, London, W.C., by the *first post on Thursdays*, and are not addressed PRIZE EDITOR, will in future be disqualified and disregarded.

N.B.—Each paper must be signed by the author with his or her real name and address. A *nom de plume* may be added if the writer does not desire to be referred to by us by his real name. In the case of all prize-winners, however, the real name and address will be published.

## Figures and Tables

**Figure f1:**
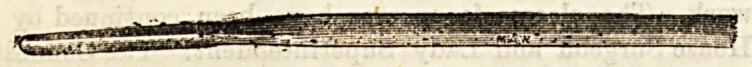


**Figure f2:**
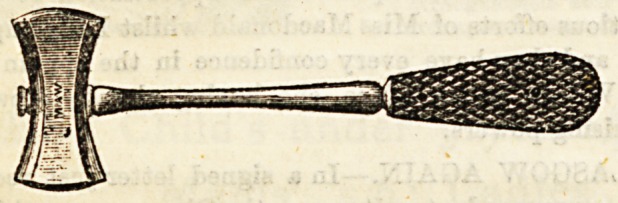


**Figure f3:**
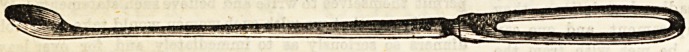


**Figure f4:**
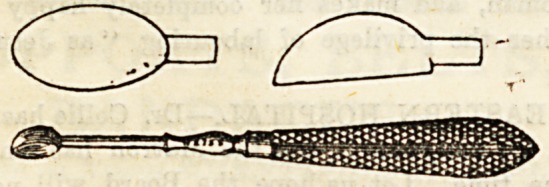


**Figure f5:**
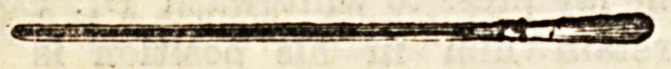


**Figure f6:**
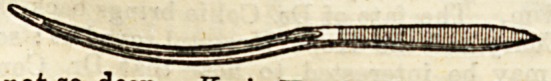


**Figure f7:**
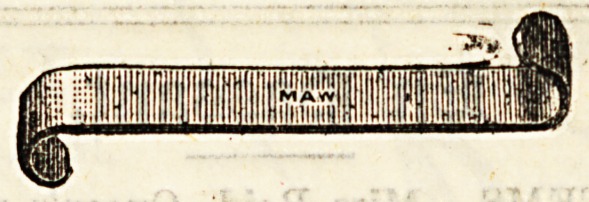


**Figure f8:**
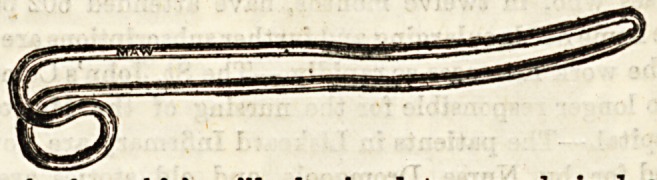


**Figure f9:**
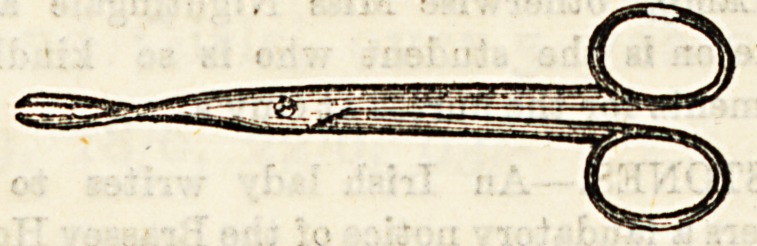


**Figure f10:**
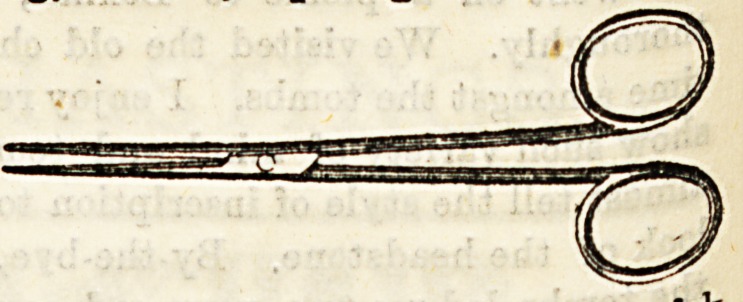


**Figure f11:**